# Saudi Nurse Interns’ Experiences during the COVID-19 Pandemic: A Thematic Approach

**DOI:** 10.3390/healthcare11020230

**Published:** 2023-01-12

**Authors:** Ejercito Mangawa Balay-odao, Jonas Preposi Cruz, Abdulellah M. Alsolais, Junel Bryan Bajet, Nahed Alquwez, Ahmed Mansour Almansour, Khalaf Aied Alotaibi, Jennifer Mesde, Ahmed Almoghairi, Bader A. Alrasheadi, Jazi Shaydied Alotaibi

**Affiliations:** 1 Department of Medicine, School of Medicine, Nazarbayev University, Astana City 010000, Kazakhstan; 2 School of Advanced Studies, Saint Louis University, Baguio City 2600, Philippines; 3 Department of Nursing, College of Applied Medical Sciences, Shaqra University, Al-Dawadmi 11961, Saudi Arabia; 4 Department of Nursing, College of Applied Medical Sciences, Majmaah University, Al-Majmaah 11952, Saudi Arabia

**Keywords:** COVID-19, nurse interns, experiences, Saudi Arabia, thematic approach

## Abstract

Background: The learning process for nurses, including internships, was affected during the COVID-19 pandemic, which may have made the nurse internship program more challenging and stressful for participants. Therefore, it is significant to explore the experiences of nurse interns during COVID-19. Aim: This study aimed to explore Saudi nurse interns’ field experiences during the pandemic. Design: The study utilised descriptive phenomenological qualitative research and a thematic approach. Methods: A total of 19 nurse interns participated in the study, which was conducted in Saudi Arabia. Participants undertook an internship program at different government hospitals in five cities in Saudi Arabia. Unstructured individual interviews were conducted to gather data from the participants. Results: The findings revealed five themes: being passionate, lacking knowledge and skills, being concerned about their families, being cautious, and being unoriented. Conclusion: The study findings document that the struggles of nurse interns in their internship programs during COVID-19 were related to their lack of knowledge, their family, and the working environment.

## 1. Introduction

Nursing internships help future nurses to become more professional, expand their network, and gain clinical knowledge while providing them with practical patient care experience. For nursing students, an internship is essential to bridge the knowledge gap between theory and practice [1]. It is noted that successful nursing internship programs integrate teaching and learning, and these have received recognition for their capacity to help recent graduates transition from novices to advanced beginners who can demonstrate their ability to perform according to established standards and handle real-world situations [2]. The importance of applying nursing principles in the clinical setting was emphasised by Florence Nightingale’s theory of nursing [3]; therefore, nursing practice requires that student nurses spend a substantial number of clinical hours putting their knowledge and abilities to use in clinical care. In addition, many students have stated that they learned best by observing real-world situations and applying what they had learned from their respective nursing schools [4].

The nurse internship program allows nurse interns to practice and improve their skills while they are supervised by staff nurses, head nurses, and nurse educators [5]. Furthermore, nursing internships allow students to develop competencies and put theory into practice, ultimately leading to the students’ incorporation into the professional world. Internships allow students to face new challenges and put their knowledge and skills to the test. They also emphasise the value of person-centred care, clinical reasoning, and reflective practice as motivators for student learning. Besides, because students have clinical and academic tutors with whom they can share their experiences, they can manage the various emotional challenges that internships present [5]. Finally, internships can influence in which healthcare field a student chooses to specialise. In Saudi Arabia, the nurse internship is a one-year program implemented in the final year of training, after the nurse completes the academic courses. The internship is organised and monitored by the university, while internship training is provided by the internship facilities [6]. During the year, interns rotate through different areas, such as emergency, intensive care, medical-surgical, paediatric, and psychiatric departments, as well as primary care clinics. Moreover, this internship program for student nurses prepares them to accept their future role as staff nurses [7] and leads to self-improvement and work commitment [8].

Nursing internships take place in healthcare settings, and clinical learning environments were severely impacted by the COVID-19 outbreak. Learning possibilities for students were impacted, since clinical placements were halted, universities closed, and in-person courses replaced with online instruction [9]. The pandemic shifted students’ learning from regular face-to-face to online platforms, such as learning management systems (LMS), Google Scholar, and Zoom meetings. While lessons and courses swiftly transitioned to online learning in order to protect students’ education and faculty activity, it was not possible to manage pre-clinical activities, such as simulations and labs, to support technical and relational competencies. Most importantly, it was not possible to set up clinical placements because of the unpredictability of healthcare environments and the social and organisational constraints put in place to prevent unwarranted access to services [10]. The pandemic created many changes in traditional internship programming in hospitals [11]. This delay and the changes in the internship program owing to COVID-19 made the program more challenging and stressful for nurse interns. A delay in being accepted affects the learning process of the nurse interns, and it was only in early 2021, after 3 months’ suspension, that most hospitals in Saudi Arabia started to accept nurse interns again. Thus, research on the experience of nurse interns during the pandemic is necessary to understand their learning experiences and struggles.

Many scholars have studied the impact of the pandemic on the mental health of interns. Findings suggest that student nurses experienced more stress during the pandemic, particularly during their practical and internship programs in hospitals [12]. Another source of stress for student nurses was their inability to perform their clinical practices [13]. In addition, the pandemic had a negative emotional impact, particularly regarding depression and anxiety, on higher education students [14,15,16]. However, as studies in Saudi Arabia exploring the experience of nurse interns during the pandemic are limited, the current research was conducted to explore such experiences.

During the pandemic, nursing schools needed to suspend classes and internship programs to decrease the risk that students and patients would contract COVID-19 [17], a situation that compromised student nurses’ learning [18]. Although online classes were conducted, opportunities for internship experiences were not available, thus denying students the opportunity to gain essential hands-on experience. In early 2021, the internship program was re-established in Saudi Arabia. However, the nurse interns’ safety and perceptions of their safety were questioned. Questions concerning safety were of greatest concern to nursing interns during the pandemic. As Eweida et al. [19] mentioned, nurse interns were in a dilemma over whether to provide care for their patients or think of their own safety first. In Great Britain, nursing students could choose to provide care for COVID-19 patients or leave their internship workplace [20]. Although the training for nurse interns generally makes them feel safe and secure in their clinical internship program [21], if their experiences are overwhelming and drain their focus and energy, the impact can be strong. Difficult experiences can lead to burnout and work-related stress, which are the most common concerns of nurse interns [22]. These outcomes can, however, be eased by the development of self-worth, personal rewards, and self-development [23].

The internship program is beneficial to student nurses, as it helps them develop creativity, skills, analytic power, and adaptability to work [7]. Furthermore, nurse interns improve their professional and individual abilities and work engagement during the internship program [8]. Although some studies have explored the experience of nurse interns during COVID-19, such studies in Saudi Arabia are limited; thus, the research question, ‘What was the experience of nurse interns during the COVID-19 pandemic in Saudi Arabia?’ was formulated to fill this gap.

## 2. Materials and Methods

### 2.1. Design

The study utilised descriptive phenomenological qualitative research and a thematic approach [24] to explore nurse interns’ experiences during the pandemic.

### 2.2. Sample

The data were collected from 19 participants from the three campuses of Shaqra University (the Shaqra main campus, Alquyiyah, and Dawadmi). These participants had been in their internship program for 6 to 12 months. The participants were chosen using the purposive sampling technique, based on their availability and willingness to participate. The participants had to have completed at least 3 months of their internship program; thus, nurse interns who were new to the program were excluded. Data saturation was achieved after 19 participants had been interviewed. According to Lincoln and Guba [25], data saturation is achieved when no new data emerge, and according to Morse [26], is ‘the most frequently invoked guarantee of qualitative rigor’ (p. 587). The saturation point is based on the number of participants, and Hennink, Kaiser, and Marconi [27] state that it can be achieved with 16–24 interviews.

### 2.3. Data Collection

Unstructured individual interviews were conducted to gather data from the participants from December 2021 to February 2022. The unstructured approach included asking core questions which allowed participants to freely share their experiences of being nurse interns during the pandemic. The interviews were carried out face-to-face, based on the preferred time and place of the participant. Most of the interviews were conducted in the university, a private room in a coffee shop, or a non-crowded coffee shop, to avoid distraction. Participants were asked to describe their internship experiences during the pandemic, after which follow-up questions were asked to further explore their experiences. Each participant interview lasted from 40 to 60 min. After the data collection, the researchers listened to the audio recording, placed data in the field notes, coded, manually extracted phrases, and developed themes. An external qualitative researcher validated the data for accuracy.

### 2.4. Ethical Considerations

The Scientific Research Ethics Committee, Shaqra University, Saudi Arabia (Ethics Approval No. ERC_SU_20210044, dated 2 November 2021) ensured the ethical conduct of the study and confirmed that written consent was given before the study was carried out. Voluntary participation in the study was ensured. Nurse interns were given code names to protect their identities. In ensuring confidentiality, the researchers made sure that no phrases or words would associate the nurse interns with their identity and internship workplace. In addition, the data were not shared and were kept in a password-protected computer for privacy purposes. Finally, the researcher obtained consent to record the interview from each nurse intern who participated in the study. An external evaluator was allowed to listen to the recording to check the accuracy of the data. Then, the recording was deleted after the study concluded.

### 2.5. Data Analysis

Following the thematic approach, the researchers were guided by the following steps when analysing the data: (1) data acquaintance or familiarisation, (2) data coding, (3) determining patterns among the codes and developing initial themes, (4) reviewing the themes by rereading the dataset and comparing the themes against the data, (5) naming and defining themes, and (6) writing the results of the thematic analysis [24,28].

After transcribing the data, each researcher manually coded all the transcript files. The 80 codes extracted from the data were narrowed down to 10, after which themes were developed. Five themes were identified in this study, reflecting the experience of nurse interns during the COVID-19 pandemic.

### 2.6. Rigour/Trustworthiness

Credibility, dependability, confirmability, transferability, and reflexivity criteria were observed to ensure the rigour and trustworthiness of the study [25]. To ensure the credibility of the data, the researcher read and reread the individual transcripts to understand the patterns and similarities in the experiences of the nurse interns. Moreover, an external evaluator was asked to review the individual transcript files and the results of the study. Participants were also asked to review the codes and themes extracted to exemplify their experiences. The researchers applied reflexivity, member checking, peer examination, and peer debriefing in the data analysis. Furthermore, the external evaluator helped the researchers express their feelings regarding the internship program during the pandemic to remove their personal bias and ensure that their personal opinions would not affect the results of the study.

## 3. Results

Participants were aged from 22 to 27 years old, and most were male [12]. Most of the student clinical area assignments were the emergency room (ER), medical ward, renal unit, surgical ward, intensive care units (ICU), obstetric and paediatric ward, and outpatient department (OPD) (see Table 1).

This study aimed to explore the experiences of nurse interns during the COVID-19 pandemic. The data were manually extracted by the researchers. Five themes were identified in this study reflecting the experience of nurse interns during the COVID-19 pandemic. These themes are being passionate, lacking knowledge and skills, being concerned about their families, being cautious, and being unoriented.

### 3.1. Being Passionate

The participants mentioned that they should be passionate about providing patient care to ensure quality nursing care, regardless of a patient’s condition and the nature of the disease. Moreover, the participants viewed caring for another person as their calling from ‘Allah,’ which made them more passionate about their role and responsibilities as future nurses:

*“It makes my desire, really and honestly, stronger, especially to be a nurse. During this time, I read more about the current situation, which increases my awareness, and I feel like there is a need to help others and my ‘Allah’ calling me to do that. I am moved by people getting sick.” NI 12*

Generally, this pandemic did not affect the desire of the nurse interns to become nurses, but it made them realise that being a nurse is a calling and a passion:

*“My desire to become a nurse during this pandemic didn’t change; in fact, it motivated me to become a better nurse to help COVID patients or patients get the best help possible.” NI 14*

This also seems to enhance their role of being good communicators and educators for patients and their families. They seemed to be aware that such communication and education were essential while caring for patients and their families. One of the students reported that:

*“Communication when treating COVID patients is very important. They are depressed already. So, we need to treat them properly and communicate with them. This is good for their mental health.” NI 19*

*“I became the teacher of the family. I have to be responsible for their health. I teach them hygiene.” NI 15*

### 3.2. Lacking Knowledge and Skills

The nurse interns in this study realised that they lacked nursing knowledge and skills during their internship experiences and understood the vital need to learn about the concept of nursing. They mentioned that nursing knowledge is fundamental when dealing with or managing patients’ diseases, particularly this emerging new disease. The knowledge of how to deal with infectious diseases is vitally needed to help them manage any possible infectious disease in the future. Moreover, they ensure that they continually practice infection control for their own safety and protection:

*“This pandemic made me realise the importance of nursing knowledge since, honestly, I don’t know what I am doing in the hospital, and also my confidence is low because I know I did not take my lectures at university seriously.” N I8*

*“We just realised the importance of personal protective equipment (PPE) and hygiene. Before, we never cared about hand washing and never cared about protecting ourselves.” NI 15*

Some of the nurse interns in this study struggled to provide nursing care for their patients because of their lack of knowledge and skills, which were affected by the online classes conducted during the pandemic. Thus, as they honed their nursing knowledge and skills, they needed to show the skills they had learned at university to gain the trust and confidence of staff nurses and show them that they were able to carry out nursing procedures independently:

*“I hope I studied well because my nursing knowledge and skills are insufficient.” NI 16*

*“Inadequate clinical skill development due to online classes and lack of motivation because of this pandemic.” NI 12*

During their internship program, they noticed that staff nurses lacked trust and had high expectations, making the experience challenging and frustrating. The internship program was frustrating because, as gaining the staff nurses’ trust was difficult, they did not perform certain nursing procedures (which they were capable of performing) independently:

*“It was hard during the early days of my training because I felt and noticed that nurses didn’t allow us to do the procedures that we know we can do alone.” NI 4*

*“My confidence is affected by my lack of knowledge and skill. I need to read to cope with my training expectations since nurses expect us to provide care without orientation. But some nurses don’t trust us, and they don’t allow us to do the procedures because they don’t believe that we can do the procedures. It is hard, since we need to prove ourselves. Also, we need to follow guidelines and protocols set by the Ministry of Health about COVID-19, which is another adjustment for me.” NI 8*

### 3.3. Being Concerned about Their Families

The nurse interns struggled to deal with the psychological impact of COVID-19 on their families. Their family members were worried about their health and the risk of being infected with COVID-19. Thus, they were prevented from joining the internship program. As mentioned by a participant:

*“This pandemic makes a difference, and everyone is worried about their health. Like my family, they don’t want me to have my clinical training because they worry that I will be infected. So, I need to explain that we are protected in the hospital, and we are not yet assigned yet to the COVID unit or to handle patients with COVID-19.” NI 5*

*“I need to explain to my father that having my training is safe and the hospital provides PPE for us.” NI 3*

Nurse interns also struggled in regard to informing and persuading their family members about their safety when they joined the internship program. The stigma attached to the information on social media—that many health care providers were infected with COVID-19 in the line of duty—influenced their families’ decisions to allow them to join the internship program. Thus, providing information about the nature and scope of their work and responsibility during their training helped in easing and decreasing the psychological struggle of their family members:

*“My family is so worried…. what I did was allow me to have my training because he was worried about my health. He was worried that I would be infected during the training. Also, I might bring the virus home.” NI 2*

*“I needed to give information to my mother about my work in the hospital as a trainee. I needed to tell her that I would not be allowed to care for patients with COVID-19, which was true during the early days of our training, but eventually, when we were used to our work, we were allowed to provide care at the ICU where COVID-19 patients are admitted. A lot of explanations were made before I was allowed to have my training.” NI 1*

### 3.4. Being Cautious

During their internship experiences, some nurses were more cautious while caring for patients. They appeared to be careful about controlling environmental hazards and eliminating infection. For example, nurse interns followed environmental health practices, such as observing social distancing when providing care to suspected cases, disinfecting their environment, and ensuring they did not inhale contaminated air by using face masks:

*“I realised that washing hands and disinfecting tables and objects exposed to a patient with COVID-19 is important. Also, I maintained social distancing if a patient was suspected of COVID-19 and used PPE to protect myself. Also, I always used face masks for my protection.” NI 2*

*“I practice my infection control, like hand washing and the use of PPE. I know it will protect me from getting infected. So, I’m not scared to care for a patient with COVID-19.” NI 17*

Nurse clinical interns became vigilant in performing their tasks by practicing infection control. In addition, their awareness of the need to prioritise patients’ safety and a safe working environment was enhanced:

*“I am cautious about infection matters. I make sure that I clean my hands before and after contact with a patient because I am afraid to transfer the virus between patients. After all, it will be my fault if they have an infection. When dealing with patients, I also avoid touching used equipment to minimise infection and protect myself and other patients. I also take standard precautions even if they are not known to have an infection.” NI 13*

### 3.5. Being Unoriented

Another problem encountered by the participants during their internship program was the lack of proper orientation in regard to their duties and responsibilities because the hospitals were understaffed. Staff nurses were busy with their responsibilities, and the interns’ learning was sometimes compromised because no staff nurses were assigned to supervise them:

*“Due to understaffing in the hospital, I did not know what our role was in the hospital. The tasks were not well explained. No proper orientation was done.” NI 15*

## 4. Discussion

This study aimed to explore nurse interns’ experiences during the pandemic in Saudi Arabia. In the present study, nurse interns stated that they lacked knowledge and skills during their internship. This finding contradicts a study conducted in Ireland, which showed that most nursing interns were prepared for their clinical duties [29] and indicated that the pandemic affected student nurses’ learning. The lack of knowledge among students was due to the sudden shift from regular face-to-face classes to online classes. The participants mentioned that they struggled to adjust and cope with the online classes, primarily because practical lectures were conducted via platforms such as Google Meet, Zoom, and LMS, which they found less helpful because they participated in them from settings within their ‘comfort zones,’ such as in their homes, in the car, or in a coffee shop, where it was difficult to concentrate and focus on their learning. These issues were a global challenge for student nurses, affecting their concentration and ability to understand their lectures [30].

Another main theme of this study is being cautious. The uncontrollable spread and deadly nature of COVID-19 caused health problems and stress to individuals, which increased the precautionary measures they took. Most of the participants mentioned that their knowledge and skills regarding infectious control improved. Similarly, a study in the United States showed that, during the pandemic, most people practiced preventive control concerning COVID-19, such as disinfecting and handwashing [31].

In addition, nurse interns took part in the measures to prevent the spread of the pandemic while providing care for their patients by observing the precautionary measures implemented by the World Health Organisation (WHO) and Ministry of Health, Saudi Arabia. Moreover, the perspective of the nurse interns on infection control measures changed, and these measures were considered essential to prevent the spreading of the disease and control the pandemic. Similarly, a study conducted after the COVID-19 pandemic revealed that individual preventive measure behaviours had increased [32]. Thus, nurse interns realised that the preventive measures imposed during the COVID-19 pandemic were advocated in Nightingale’s theory of nursing. Florence Nightingale advocated that health practitioners should help maintain a person’s well-being and basic health needs by preventing disease transmission [33]. In addition, Nightingale mentioned that disease prevention began at home [34]. Thus, nurse interns played their part by educating their family members.

Another finding of the study is the impact of understaffing during the pandemic. Understaffing was one of the main challenges to any organisation during the pandemic [14]. Nurse interns struggled with their learning process and to hone their skills because of the lack of staff nurses to provide supervision and orientation. This problem had a domino effect on nurse interns: the learning of the nurse interns, whereby they become competent future nurses, was compromised, considering that mentorship is essential in developing the knowledge and skills of nursing students [13].

The willingness of the nurse interns to undertake their internship program, even though their health was at risk, was impacted. The nurse interns’ willingness to provide and ensure quality care fuels their desire to work. Similarly, a study in the United States showed that student nurses had a high degree of willingness to provide nursing care in hospital settings [33]. Thus, this willingness of nurse interns helps them to view their role as fulfilling and rewarding. This behaviour comes naturally for Muslims, as they love to maintain close relationships with their friends, family, and Allah [35].

## 5. Conclusions

The study findings document that nurse interns understand the importance of infection control and their role as educators and communicators, as well as the significance of nursing as their calling and passion. Moreover, the struggle of nurse interns in their internship programs during COVID-19 was related to their lack of knowledge, their families, and the working environment. In addition, nurse interns struggled with the stigma of information posted on social media and viewed by their families and the impact of online classes on their knowledge and skills. They also experienced a lack of trust and high expectations from staff nurses and the effect of understaffing on their role as nurse interns.

The study findings imply the need for nursing education and training hospitals to intensify the theoretical and practical learning of nursing students before allowing them to undertake their clinical practice in internship programs. The training institution should ensure that they have planned appropriate activities for nurse interns. Furthermore, mentorship programs in the hospital should be improved to ensure that a nurse is assigned to each nurse intern to follow up their activities and augment learning needs. Training hospitals should ensure the availability of a training officer that can supervise nurse interns to maximise their learning. Nursing schools should also monitor and evaluate the activities of students in the different learning facilities and delegate a clinical coordinator to visit nurse interns at least once a month for monitoring, evaluation, and coordinating their learning needs. In regards to practical courses, universities should ensure that the materials used in online learning, such as videos and case analysis studies, offer ideal nursing knowledge that student nurses can put into practice. It should be noted that the study did not focus on the impact of mentorship on the learning experience of the nurse interns or on the types of leadership skills among mentors; thus, future researchers should consider these aspects.

## 6. Limitations of the Study

The study utilised unstructured interviews; in some cases, nurse interns may have been hesitant to disclose information regarding their internship for fear that their internship program and performance evaluation would be affected. This limitation was addressed by the researchers’ ensuring that no information or data would be associated with the participants and by randomly choosing participants from different nursing schools using a referral system. In addition, as this a qualitative study, the sample was small and was taken from one university, which is not representative of the nursing experience across Saudi Arabia.

## Figures and Tables

**Table 1:** Profile of the Participants.

Participants	Age	Gender	Months as Nurse Intern	Area of Clinical Assignment
Nurse intern (NI) 1	22	Female	8	ER, Renal Unit, Obstetric and Paediatric Ward
Nurse intern (NI) 2	23	Female	8	ER, Renal Unit, Surgical Ward
Nurse intern (NI) 3	23	Female	8	ICU, Medical Ward, Obstetric and Paediatric Ward
Nurse intern (NI) 4	23	Male	7	ER, Renal Unit, Medical Ward, Surgical Ward
Nurse intern (NI) 5	23	Male	8	ER, Surgical Ward, ICU, Medical Ward
Nurse intern (NI) 6	22	Male	8	ER, Renal Unit, Medical Ward, ICU
Nurse intern (NI) 7	23	Male	7	ER, Renal Unit, Surgical Ward
Nurse intern (NI) 8	25	Male	8	ER, Surgical Ward, ICU, Medical Ward
Nurse intern (NI) 9	23	Male	8	ER, Surgical Ward, ICU, Medical Ward
Nurse intern (NI) 10	23	Female	8	ER, Renal Unit, Obstetric and Paediatric Ward, ICU
Nurse intern (NI) 11	23	Female	8	ER, Renal Unit, Surgical Ward, Obstetric and Paediatric Ward
Nurse intern (NI) 12	23	Female	8	ER, Surgical Ward, ICU, Obstetric and Paediatric Ward
Nurse intern (NI) 13	25	Female	8	ER, Renal Unit, Surgical Ward, Obstetric and Paediatric Ward
Nurse intern (NI) 14	24	Male	9	ER, Surgical Ward, ICU, Medical Ward
Nurse intern (NI) 15	25	Male	6	ER, Renal Unit, Surgical Ward
Nurse intern (NI) 16	25	Male	9	ER, Surgical Ward, ICU, Medical Ward
Nurse intern (NI) 17	26	Male	9	ER, Renal Centre, Surgical Ward
Nurse intern (NI) 18	27	Male	12	ER, Renal Unit, Surgical Ward, Medical Ward, ICU, OPD
Nurse intern (NI) 19	26	Male	12	ER, Renal Unit, Surgical Ward, Medical Ward, ICU, OPD

## Data Availability

Data is available upon request.
